# Supplementary material for: Quantitative Multiplex Real-Time Reverse Transcriptase–Polymerase Chain Reaction with Fluorescent Probe Detection of Killer Immunoglobulin-Like Receptors, KIR2DL4/3DL3
Source: Diagnostics (Basel). 2020 Aug 13;10(8):588. doi: 10.3390/diagnostics10080588 (PMC7460478; doi:10.3390/diagnostics10080588)
Supplement: Supplementary file 1 [file diagnostics-10-00588-s001.zip › diagnostics-892504-supplementary.pdf]

Table 1. Total number of alleles of 17 KIR genes.

KIR Gene	Number of Alleles	KIR Gene	Number of Alleles
KIR2DL1	111	KIR2DS4	39
KIR2DL2	34	KIR2DS5	24
KIR2DL3	64	KIR2DP1	40
KIR2DL4	109	KIR3DL1	184
KIR2DL5A	21	KIR3DL2	165
KIR2DL5B	37	KIR3DL3	166
KIR2DS1	17	KIR3DS1	39
KIR2DS2	24	KIR3DP1	29
KIR2DS3	16		

Table 2. The percent identities between mRNA nucleotide sequences of KIRs.

.	2DL1*001	2DL2*0010101	2DL3*0010101	2DL4*00101	2DL5A*0010101	2DS1*001	2DS2*0010101	2DS3*00101	2DS4*0010101	2DS5*001	3DL1*0010101	3DL2*0010101	3DL3*00101
2DL2*0010101	96.94												
2DL3*0010101	95.80	97.75											
2DL4*00101	77.64	77.15	77.95										
2DL5A*0010101	79.88	79.79	80.44	88.12									
2DS1*001	96.38	94.41	95.08	75.67	77.12								
2DS2*0010101	94.74	96.82	97.49	76.23	78.57	96.50							
2DS3*00101	93.64	93.75	94.21	74.48	76.90	95.85	95.85						
2DS4*0010101	93.20	93.20	93.77	76.67	78.12	95.52	95.63	93.77					
2DS5*001	93.86	92.98	93.88	74.89	77.01	95.74	95.08	96.72	93.44				
3DL1*0010101	88.25	88.35	88.30	75.02	76.82	85.57	86.99	84.92	85.68	84.81			
3DL2*0010101	87.20	86.72	87.33	75.14	76.50	85.03	85.79	84.70	85.46	84.37	93.03		
3DL3*00101	83.81	83.70	84.70	72.63	73.95	82.41	83.10	80.90	82.75	81.37	86.70	87.43	
3DS1*010	84.59	84.82	85.10	70.69	72.11	84.64	86.12	84.30	84.76	84.07	97.34	91.32	84.64

Table 3. The reproducibility and cross reactivity assessment.

**Template DNA copies**	**KIR2DL4**			**KIR3DL3**			**RPII**		
	**Mean**	**SD**	**%CV**	**Mean**	**SD**	**%CV**	**Mean**	**SD**	**%CV**
Singleplex qRT-PCR (*n* = 6)									
5×10^1	37.91	0.80	2.11	41.79	2.68	6.41	37.08	0.70	1.89
5×10^2	35.24	0.54	1.55	35.26	0.45	1.29	33.80	0.74	2.19
5×10^3	32.01	0.63	1.96	32.50	0.42	1.30	30.08	0.34	1.12
5×10^4	29.39	0.63	2.14	29.17	0.44	1.52	26.54	0.18	0.66
5×10^5	25.59	0.31	1.20	26.02	0.59	2.26	23.77	0.24	1.01
5×10^6	22.30	0.61	2.72	22.92	0.69	3.00	20.68	0.26	1.26
5×10^7	18.81	0.39	2.08	19.46	0.86	4.40	17.06	0.40	2.37
5×10^8	16.11	0.57	3.57	16.14	0.80	4.96	14.36	0.10	0.72
Irrelevant plasmid	44.15	1.12	2.53	44.06	1.72	3.91	44.12	1.44	3.27
Cross-reactivity (*n* = 3)									
$5x10^7$ KIR2DL4	-	-	-	37.00	0.54	1.46	44.67	0.53	1.20
$5x10^7$ KIR3DL1	36.44	0.65	1.78	35.76	0.25	0.69	44.65	0.88	1.97
$5x10^7$ KIR3DL3	37.50	0.52	1.38	-	-	-	45.01	0	0
$5x10^8$ RPII	44.72	0.70	1.57	44.94	0.16	0.36	-	-	-

Mean; Mean of Cycle Time (C_T); SD; Standard deviation; % CV; The percentage of Coefficient of Variation (between run).

Table 4. Reproducibility assessment of reference gene in multiplex assays.

Template DNA	KIR2DL4			KIR3DL3			RPII		
**Copies**	**Mean**	**SD**	**%CV**	**Mean**	**SD**	**%CV**	**Mean**	**SD**	**%CV**
n = 3 (0.4 μM primer and probe)							**($5x10^2$) RPII**		
5×10^2	44.99	4.10	9.11	43.61	4.55	10.43	33.98	0.21	0.62
5×10^3	31.28	0.95	3.04	32.11	0.80	2.49	33.99	0.29	0.85
5×10^4	29.28	0.46	1.57	28.45	1.09	3.83	34.00	0.15	0.44
5×10^5	25.81	0.93	3.60	26.00	0.91	3.50	45.01	0	0
5×10^6	22.34	0.31	1.39	23.11	0.61	2.64	45.01	0	0
n = 3 (0.8 μM primer and probe)							**($5x10^2$) RPII**		
5×10^2	42.29	3.63	8.58	42.37	3.28	7.74	33.95	1.34	3.95
5×10^3	31.85	0.75	2.35	31.52	1.01	3.20	33.37	0.65	1.95
5×10^4	29.46	1.26	4.28	28.54	0.88	3.08	33.63	0.62	1.84
5×10^5	25.79	0.92	3.57	25.77	0.68	2.64	45.01	0	0
5×10^6	22.47	0.98	4.36	22.80	0.49	2.15	45.01	0	0
n = 6 (0.4 μM primer and probe)							**($5x10^3$) RPII**		
5×10^2	44.29	2.26	5.11	44.11	2.84	6.43	29.54	0.43	1.47
5×10^3	31.67	0.49	1.55	31.60	0.26	0.81			
5×10^4	29.11	0.46	1.59	28.72	0.53	1.84			
5×10^5	25.38	0.46	1.82	25.91	0.59	2.27			
5×10^6	22.27	0.57	2.55	22.55	0.53	2.33			

Mean; Mean of Cycle Time (C_T); SD; Standard deviation; %CV; The percentage of Coefficient of Variation (between run).

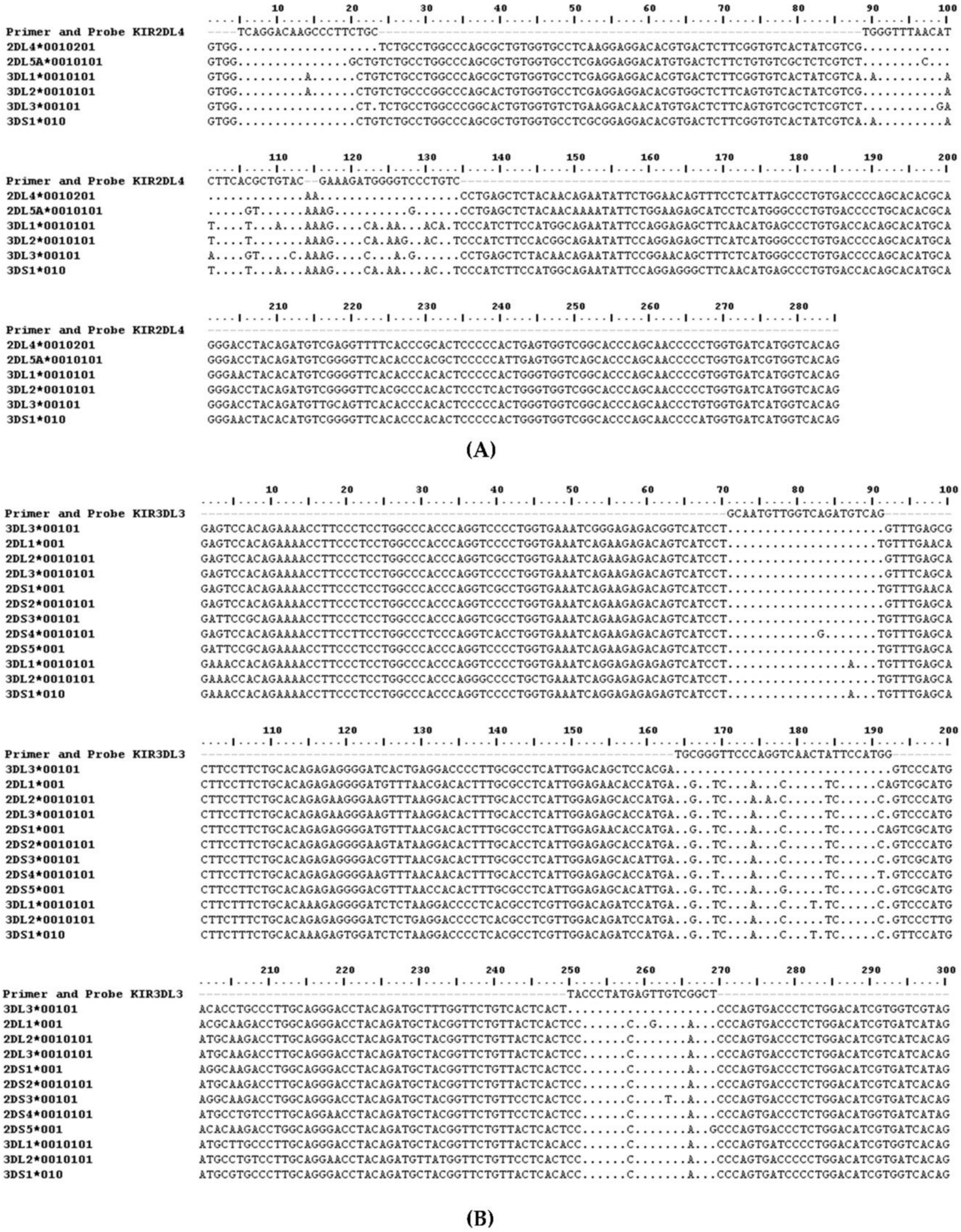

Figure 1. Comparative multiple-sequence alignments of primer and probe sequences. Primers and probes for KIR2DL4 (**A**) and KIR3DL3 (**B**) were aligned with mRNA nucleotide sequences of other KIRs retrieved from the IPD Database using the BioEdit version 7.0.4. Primers and new probes designed between the forward and reverse primers in this study were shown as well as the target sequences for hybridization. Probes and primers located in exon 4 of KIR2DL4 are at the nucleotide positions 5-23, 89-113 and 116-134, and in exon 3 of KIR3DL3 at the nucleotide positions 71-91, 164-193 and 250-269, respectively. A dot represents a nucleotide identity to the reference sequence and a dash represents a gap inserted to obtain optimal alignments.

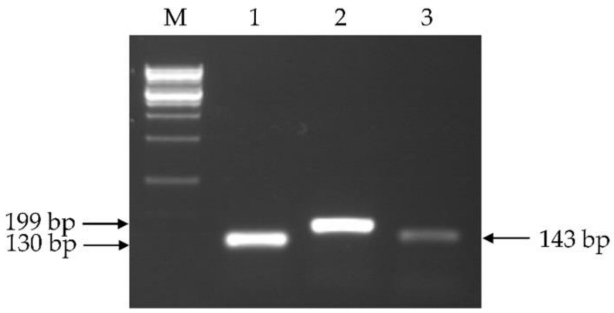

Figure 2. Amplification of segmented cDNA of KIR2DL4, KIR3DL3 and RPII by RT-PCR assay. Specific primers were used to generate the 130, 199 and 143bp fragments specific to KIR2DL4, KIR3DL3 and RPII, respectively. M represents the 100 bp DNA ladder. Lane 1: segmented cDNA of KIR2DL4, lane 2: segmented cDNA of KIR3DL3 and lane 3: segmented cDNA of RPII.

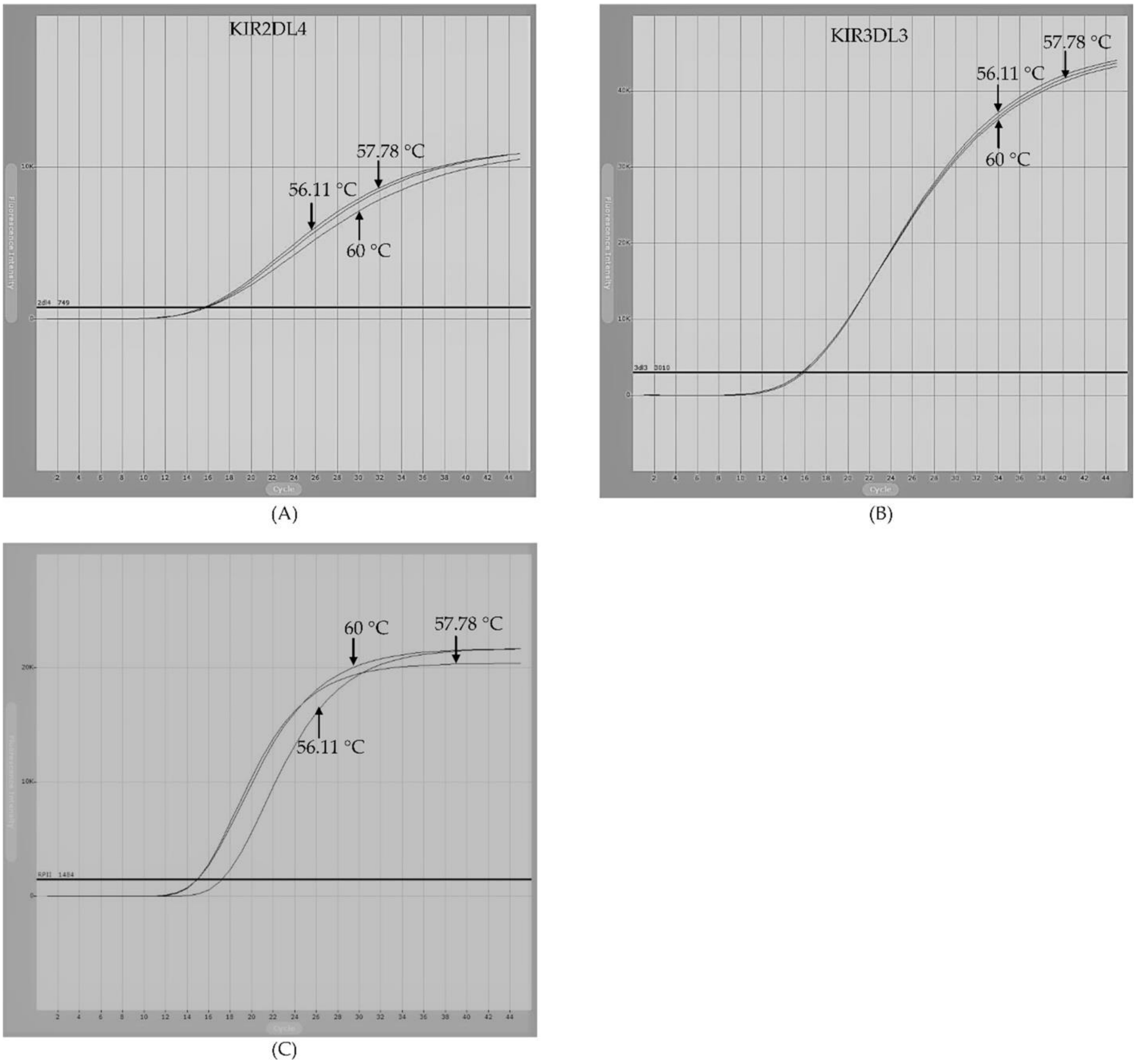

Figure 3. The optimization with varying annealing temperature for qRT-PCR. Standard plasmids (10^8 copies/reaction) were performed with primers and probes of KIR2DL4 (**A**), KIR3DL3 (**B**) and RPII (**C**) at different temperature of 56.11, 57.78 and 60 °C by real-time PCR.

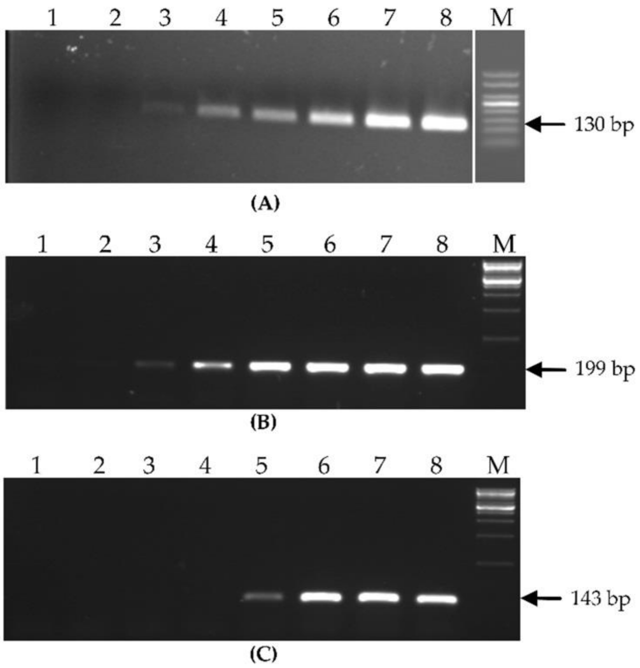

Figure S4. The sensitivity of conventional end-point PCR assay for the serial dilutions of standard plasmid DNA. Specific primers for KIR2DL4 (**A**), KIR3DL3 (**B**) and RPII (**C**) were used to amplify the standard plasmid DNA in ten-fold dilution series. Lane 1-8: standard plasmid ranged from 101 to 108 copies/reaction. M represents the 100 bp DNA ladder except 50 bp ladder for KIR2DL4.

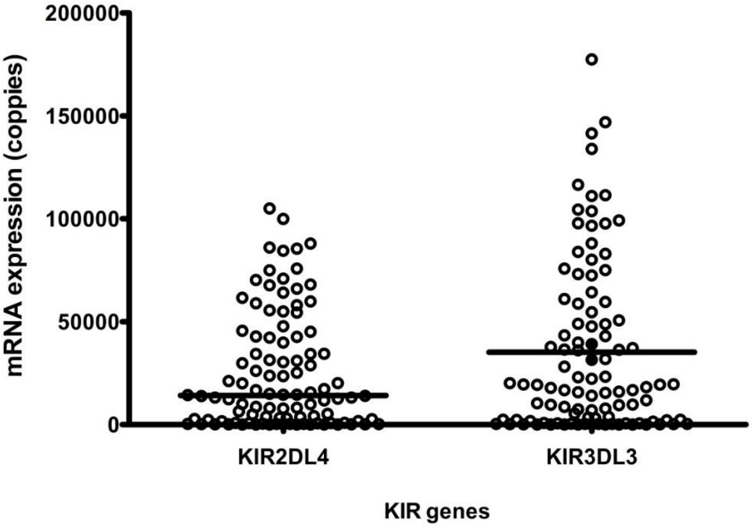

Figure 5. KIRs gene expression on clinical samples. A schematic showed frequencies of KIR2DL4 and KIR3DL3 expression in the 100 healthy donors. The data showed as the median copy numbers.
